# Syphilitic Disease of the Tongue

**Published:** 1892-08

**Authors:** C. H. Darling


### THE DENTAL REGISTER.

VOL. XLVI.] AUGUST, 1892. [No. 8.

### COMMUNICATIONS.

### Syphilitic Disease of the Tongue.

BY C. H. DARLING, M.D.

The lesions in syphilis, which may appear upon the tongue, are quite distinct and confined to no period of the disease. It is rarely the seat of the primary lesion, because the accidental transmission of virus to this locality rarely happens. When found upon the tongue, it is near the tip. It is hard, circumscribed, and quite prominent, varying in size from a small pea to a large bean. The base is sloping and rounded, while the lymphatic glands beneath the jaw are hard and enlarged. The sore is quite superficial and free from pain; it differs little in color from the surrounding tissue, and has a slight watery discharge. The primary lesion pursues much the same course here that it does in other localities, unless it is irritated, then it becomes inflamed, the discharge increases, and its course is prolonged beyond the usual period.

The lesions of the secondary stage are far more common, and are of two kinds. Those which are formed by injury or breaking down of mucous patches, the others formed by breaking down of muscular and connective tissues, or by irritation by diseased teeth.

Tubercule of mucous membrane may be sometimes taken for syphilis, but may be recognized by the portions of tuberculous tissues remaining behind, not fully destroyed by ulceration.

Ulceration of the tongue will be limited in its course according to the constitutional condition of the patient, or the amount of irritation present. The ulcers not only increase in area but become deeper. The deeper an ulcer becomes, the more clean cut will be the edges, and more unhealthy its appearance. The surrounding tissues may be thickened and no healthy granulations appear until treatment has been carried on for some time. Whatever may be the cause of these ulcers they show little tendency to inflammation. The inflammation is slight, indeed, when compared with ulcerative conditions in other than syphilitic persons. The second variety appears as small excoriations on the upper portion of the tongue, at the tip or edges. They will present no sign at first that they belong more to syphilis than to other constitutional diseases; there will be no inflammation, only one or two slight cracks or fissures. In all of these lesions syphilis may be looked upon as the predisposing cause, while irritation by the teeth is the exciting cause.

Ulcers of the secondary stage may remain a long time stationary as regards their growth, or they may slowly increase in size; they may remain for months the same, being neither better nor worse. Though not painful, they may be extremely sensitive; this together with the slight tendency toward recovery may cause the patient much uneasiness, and are constant reminders to the possessor that he has syphilis.

How can one determine that the ulcers on the tongue are due to syphilis? Sometimes this will be easy, at other times quite difficult. When the ulcer is formed by breaking down of tubercule, a portion of it remaining behind, the diagnosis is easy. In syphilis the skin lesions or enlarged, lymphatic glands may be present, at the same time, frequently there are lesions in other parts of the mouth; then there is the characteristic absence of pain and inflammation so common to syphilis. It must not be forgotten that disease of the teeth may cause similar lesions or may be an active cause in producing syphilitic lesions. Sometimes the diagnosis may be made rather by the absence of other diseases than by the presence of syphilis, no other disease presents such a variety of symptoms or is so obstinate and unyielding in its course. Healing of the ulcers of the secondary stage leaves scars on the tongue, not deep or extensive, but plainly visible; they are bright outlines which preserve with marked distinctness, the shape of the ulcer as it previously existed; a written record of past syphilis to aid us in diagnosis.

As ulcers of the tertiary period of syphilis begin in gummata, it may be well to describe them as they form in the tongue. When first formed and unbroken gummata may be superficial or deep, appearing at any time in the third stage, but usually four or five years after the primary lesion. Superficial gummata are found on the dorsum, borders and tip of the tongue. They form hard points or nodes of various sizes from a pin point to a small bean, and may be felt as hard bodies beneath the mucous membrane. They are not always well defined, but fade away into the surrounding tissue. They are indolent at first, causing no pain. When first noticed they are slightly elevated above the mucous membrane. Soon this point becomes slightly reddened and the membranes get smoother than that surrounding the point of disease. They are nearly always multiple, and a large number of them may be found on the dorsum of the tongue at one time. Soon they may become redder, softer, and in the course of time they may break down, but in some cases they may remain unchanged for months according to the condition and habits of life of the patient. The deeper gummata remain unchanged longer than the superficial.

The diagnosis of gummata is not difficult. They may be situated on portions of the tongue not easily irritated, and show little tendency to break down, but when irritated ulceration begins early. A single gumma on the border of the tongue may be mistaken for carcinoma, but the age of the patient and previous history may make the matter clear. The deep gummata also vary much in size. They may be single, as large as a hazel-nut, or there may be large gummatous masses, collections of smaller ones. They are more formidable than the superficial and more destructive in their tendencies. They are found in any part of the muscular portion of the tongue, but are most common near the median line. They may lie at almost any depth, and when lying deep are noticed only as a slight bulging of the surface of the tongue. They can be felt as rounded tumors, distinct in outline, feeling much like a foreign body in the substance of the tongue. They are not sensitive to touch, are very indolent, sometimes remaining unchanged for years, though their natural tendency, like the superficial, is to break down and become ulcers. The mucous membrane covering them is usually smooth and normal in appearance, unless they are beginning to ulcerate. As the disease progresses the tumor becomes softer and approaches the surface. However, rapidly they may grow they retain their rounded or oval form, and as they approach the surface, fluctuation may be detected in some of the larger tumors.

When several gummata exist at the same time the tongue may be greatly swollen; instances are known where the tongue could not be kept in the mouth. Deep gummata may be mistaken for tumor, carcinoma or abscess. Innocent tumors are usually single; deep gummata multiple; innocent tumors are frequently lobulated—rare condition for gummata; or innocent tumors are clearly defined and separated from the muscular tissue of the tongue, while gummata are not.

Cancerous lumps are single, the result of irritation, and form opposite a carious tooth, while gumma may have no connection with diseased or broken teeth. Cancer comes after forty years of age; gumma may come at any age, and is usually attended by a history of syphilis; at least in all doubtful cases the history of syphilis should be carefully examined.

An abscess is more clearly defined than gumma, and diagnosis may be completed by passing an exploring needle into the tumor. Gumma may also be mistaken for a foreign body in the tongue if there has been a preceding history of injury. Unbroken gummata tend to get well if the patient is in fairly good health and the treatment is persistently carried out. When no treatment is employed they tend to form ulcers which pursue a varied course according to circumstances.

These ulcers are far more formidable than those belonging to the secondary stage. Deep fissures and extensive cicatrices form an enduring record of the disease, which may be read while the patient lives. There may be one large ulcer or there may be more, according to the number of gummata which break down.

They may be round, oval, or form deep lines or fissures. Superficial fissures may deepen and at the same time widen. They may be so numerous that they cause great deformity of the tongue. The ulcers may be well defined, sharp cut and perpendicular, or their borders may be undermined, the full extent of the fissure not being seen until the walls are separated. The surrounding parts may be swollen and slightly indurated, but free from pain. The fissures are often quite sensitive and interfere with mastication. Profuse salivation is frequently produced. They may become inflamed because of irritation, the large ones presenting quite a formidable appearance, yet they are ever chronic and indolent. When very deep there can be no mistake in diagnosis, a single fissure might be due to tubercule, but the furrowed tongue of old syphilitics is seen in no other disease.

The larger ones might be mistaken for cancerous ulcers, but ulcers of syphilitic origin have a great liking for the dorsum of the tongue, and that well back. They are more common in men than in women and may sometimes be found on the tongues of children who have inherited syphilis. There will be a history accompanying the condition, but no enlargement of the lymphatics.

Gummatous ulcers rarely heal spontaneously but may remain indolent for an indefinite period. They may also inflame, and extend in various directions, eating away a large portion of the tongue. The course they pursue will depend much on the general conditions of the patient as well as upon surroundings.

### SYPHILIS IN THE MOUTH.

An early manifestation of the secondary stage of the disease will be the appearance of mucous patches in the mouth. They are rose-colored elevations rounded or oval in form, presenting a surface which much resembles mucous membrane.

You should be familiar with these lesions above all others, for in them lies great danger to yourselves of contracting the dis ease. Sometimes these patches may not be elevated, but slightly depressed. The surface may retain its epithelium, but that is generally lost. At some period they are greyish-white, as though penciled over with silver nitrate. This covering is not easily removed; it may not cover the whole surface but remains along the border. They commonly appear at the angles of the mouth and may begin in cracks or fissures. The inner border of the lower lip is a favorite situation for these lesions, and there is no part of the mucous membrane of the mouth where they may not appear. When seated upon the tonsil they frequently ulcerate because of the constant friction in swallowing. Another point where they are frequently found is on the velum palati, the pillars of the fauces, and the inner side of the cheek near the last molar tooth. The gums are rarely affected.

These mucous patches are so large, superficial, so distinct in outline, so free from pain and irritation, that they need hardly be mistaken for any other disease than syphilis.

Erythema may be found in the mouth as well as upon the skin and is usually confined to the neighborhood of the fauces; it may be so slight as to be mistaken for a slight cold. There may be some swelling of the velum and uvula at times causing it to hang far down, so that the question of removal will be raised. This should never be done as the parts soon tend to assume their normal condition, unless there be slight erosions which end in ulceration.

When papular eruptions are present on the body they may also be found in the mouth, but they are short lived, because the mucous membrane is very thin and tends to break down. The white patches found near the angles of the mouth are seen most in smokers and are due to the constant accumulation of epithelium. They may, in time, become erosions or fissures, are persistent and obstinate, liable to recur even after the specific virus has become extinct. Gummatous tumor of the soft palate comes on insidiously and frequently does a large amount of irreparable damage. The early symptoms are scarcely noticed. The patient may, at first, think he has an ordinary cold. There will be a slight tickling of the fauces followed by difficult deglutition. When he attempts to swallow he may find that the food regurgitates through the nostrils. Soon, and with little warning, he may be deprived of the power of speech, the full round voice being changed to a nasal whisper. When he attemps to swallow the liquid portion will pass through the nose.

How is gumma of this locality formed? Between the buccal and nasal surfaces may be found a small, slightly-thickened growth seemingly composed of some deposit in the connective tissue. It has the appearance of flattened tumor, at first hard to the touch, but in time it begins to soften and fluctuates like a small abscess. Again, the infiltration may be diffuse, the whole velum is tickened, the mucous membrane becomes red and the mobility of the parts is much impaired.

The fluctuating tumor breaks or ulcerates through (it may be on one or both walls). When only one side breaks it is usually in the mouth, leaving a deep ulcerating cavity with sharply cut edges. The destructive process progresses rapidly, and complete perforation soon follows. The whole velum may in this way be soon destroyed, or only a limited portion be perforated. When there is marked destruction the voice is greatly changed, being nasal and of that pitch which the French term "duck's voice." The slight pain present in the early stages has now disappeared; swallowing is not difficult, but the food, even some of the solid portions, may come through the nose. Hearing may be impaired because of swelling and inflammation about the Eustachian tube. After a time the inflammation subsides and the tissues gradually resume their normal condition. Some of the tissue has been destroyed, hence a full recovery of the voice, or the power of conveying the food along the proper channel to the throat should not be expected. Much improvement may be effected by a properly constructed plate, such as you will find fully described in works on this subject.

Is there any difficulty in determining that ulceration of the palate belongs to syphilis? Such a result is likely to follow only two causes, syphilis and tuberculosis; the latter being a rarity when compared with the first. Treatment can do much toward limiting the destructive process of ulceration when it has once begun, and surgery may often aid in repairing the damages.

